# Challenges and Elements Hindering the Adoption of Enhanced Recovery After Surgery (ERAS) Protocols in Colorectal Surgery and Their Resolutions: A Systematic Review

**DOI:** 10.7759/cureus.63222

**Published:** 2024-06-26

**Authors:** Bolaji O Ayinde, Priyank Chokshi, Sanjeev Adhikari, Aniket Jaimalani, Artashes Yeritsyan, Ashka V Surve, Safeera Khan

**Affiliations:** 1 Faculty of Medicine, California Institute of Behavioral Neurosciences and Psychology, Fairfield, USA; 2 Medicine, California Institute of Behavioral Neurosciences and Psychology, Fairfield, USA

**Keywords:** lower gi or colorectal surgery, resolution, implemenation, adoption, : enhanced recovery after surgery (eras)

## Abstract

This systematic review focuses on the various aspects of Enhanced Recovery After Surgery (ERAS) implementations, such as the various barriers, facilitators, and the role of teamwork. A systematic search was performed in PubMed, PubMed Central (PMC), and the Cochrane Library for studies published between the years 2018 and 2023. Inclusion criteria encompassed studies assessing the various factors hindering the implementation of ERAS protocols on patients undergoing colorectal surgery. It collectively highlights the importance of a multidisciplinary approach, continuing education, supervision, and patient involvement in achieving a successful implementation. Important findings include the positive impact of a performance improvement team, audits and feedback, and patient-centered approaches in reducing hospital length of stay, reducing inflammation, and improving patient outcomes.

In addition, the study emphasizes the challenges of complete adherence to all ERAS components and suggests a simplified protocol to improve implementation. This paper also seeks to understudy the hurdles encountered during the adoption of the ERAS protocol and studies the various fundamental components of the protocol.

## Introduction and background

The Enhanced Recovery After Surgery (ERAS) is a multimodal and multifactorial pathway to optimizing perioperative management [1]. The ERAS protocol was developed by a group of European surgeons. It consists of a series of evidence-based guidelines covering the whole perioperative period [2]. This protocol has been established to reduce mortality in hospital length of stay (LOS) and improve recovery after colorectal patients [3-6]. The first ERAS protocol was drafted in the early 2000s for colon surgery initially and rectal surgery, after which other surgical specialties adopted this. The ERAS guideline for colorectal surgery was last updated in 2018. It comprises 24 core elements, including preoperative, intraoperative, and postoperative measures to be implemented [4].

Since its inception, the ERAS protocol has greatly improved the outcome of innumerable patients globally in various periprocedural settings, with over 4,000 articles published in PubMed-indexed journals attesting to this [7-9]. This protocol aims to ameliorate the postoperative inflammatory response, foster early movement, and resume gastrointestinal (GI) nutrition to lower the patient's LOS and postoperative complications [8,10]. The protocol implementation is a complex process applied by a multidisciplinary team in the hospital setting, including surgeons, anesthesiologists, nurses, nutritionists, and physiotherapists, among many others [11]. Hence, increased vulnerability to failure and constant monitoring are needed to maintain sustainability and efficacy [12]. In the absence of continuous auditing, there will be a decrease in program compliance. It has been shown that a compliance rate of greater than 70% is associated with better clinical results and improved survival rates [4,13].

There are several limitations to applying the ERAS protocol as it is time and effort-intensive. Therefore, implementing all the components can be difficult, hence a need to identify the possible 'core items' [13]. Various hospitals have adopted the ERAS protocol; however, few studies address the implementation of the overall ERAS protocol. This paper will review the various setbacks to implementing the protocol and possible core aspects of the ERAS protocol. Complete adherence to the ERAS protocol is nearly impossible due to variations in setting, healthcare provider, and patient factors; however, without full adherence, partial implementation continues to provide benefits [6,14,15].

By analyzing the existing evidence, this research paper seeks to identify methods to improve adherence to the ERAS protocols and seek ways to optimize patient outcomes.

## Review

Methodology

This systematic review followed the guidelines recommended by the Preferred Reporting Items for Systemic Review and Meta-Analysis (PRISMA) 2020 protocol [16].

Search Sources and Strategy

A comprehensive search of PubMed, PubMed Central (PMC), Medline, and the Cochrane Library for pertinent articles was done. We utilized multiple combinations of implementation, adherence, ERAS, and colorectal surgery to search all databases. In PubMed, a search strategy that included keywords and PubMed's Medical Subject Heading (MeSH) database: (("Treatment Outcome"(Mesh)) AND ("Enhanced Recovery After Surgery"(Mesh))) AND (("Digestive System Surgical Procedures/methods"(Mesh) OR "Digestive System Surgical Procedures/mortality"(Mesh) OR "Digestive System Surgical Procedures/nursing"(Mesh) OR "Digestive System Surgical Procedures/rehabilitation"(Mesh) OR "Digestive System Surgical Procedures/standards"(Mesh). Table 1 shows the databases employed and the quantities of papers identified.

**Table 1:** Search strategy and number of papers identified.

Search strategy	Database	No. of papers identified
(("Treatment Outcome" (Mesh)) AND ("Enhanced Recovery After Surgery"[Mesh])) AND (("Digestive System Surgical Procedures/methods" (Mesh) OR "Digestive System Surgical Procedures/mortality"(Mesh) OR "Digestive System Surgical Procedures/nursing" (Mesh) OR "Digestive System Surgical Procedures/rehabilitation" (Mesh) OR "Digestive System Surgical Procedures/standards" (Mesh) OR "Digestive System Surgical Procedures/trends" ((Mesh)))	Pubmed (Mesh), 2018-2023	39
Enhanced Recovery after Surgery (ERAS) Protocols AND COLORECTAL SURGERY	Cochrane Library, 2018-2023	69
(Enhanced Recovery after Surgery (ERAS) Protocols) AND (COLORECTAL SURGERY)	Pubmed Central, 2020-2023	181

Inclusion and Exclusion Criteria

Some of the most recent papers were selected and comprise articles released in the last five years, either in English or full-text English-language translation. We specifically included research papers that met the following criteria: (1) focused on colorectal surgery; (2) focused on the implementation process and barriers; and (3) were published in peer-reviewed journals.

The exclusion criteria included articles without full text, gray literature and proposal papers.

Selection Process

The selected articles were transferred to Endnote, and duplicate papers were removed. Articles were screened through titles and abstracts and independently assessed by the lead author. In addition, discrepancies were discussed with the other co-authors and resolved. Shortlisted articles underwent full-text review, and those meeting the inclusion and exclusion criteria were selected for further analysis.

Quality Assessment of the Studies

Various relevant quality appraisal tools were utilized to assess the quality and screen for bias in the shortlisted articles. The Newcastle-Ottawa tool was utilized for quality assessment for observational studies, and the Assessment of Multiple Systematic Review (AMSTAR) tool was used for systematic review. Only studies meeting the quality criteria were included.

Data Collection Process

Once the articles were selected and extracted for the systematic review, the initial outcomes were assessed alongside relevant information. The lead author autonomously extracted the data, and all authors equally participated in the finalization of the retrieved data and outcomes.

Results

Study Identification and Selection

**Figure 1:**
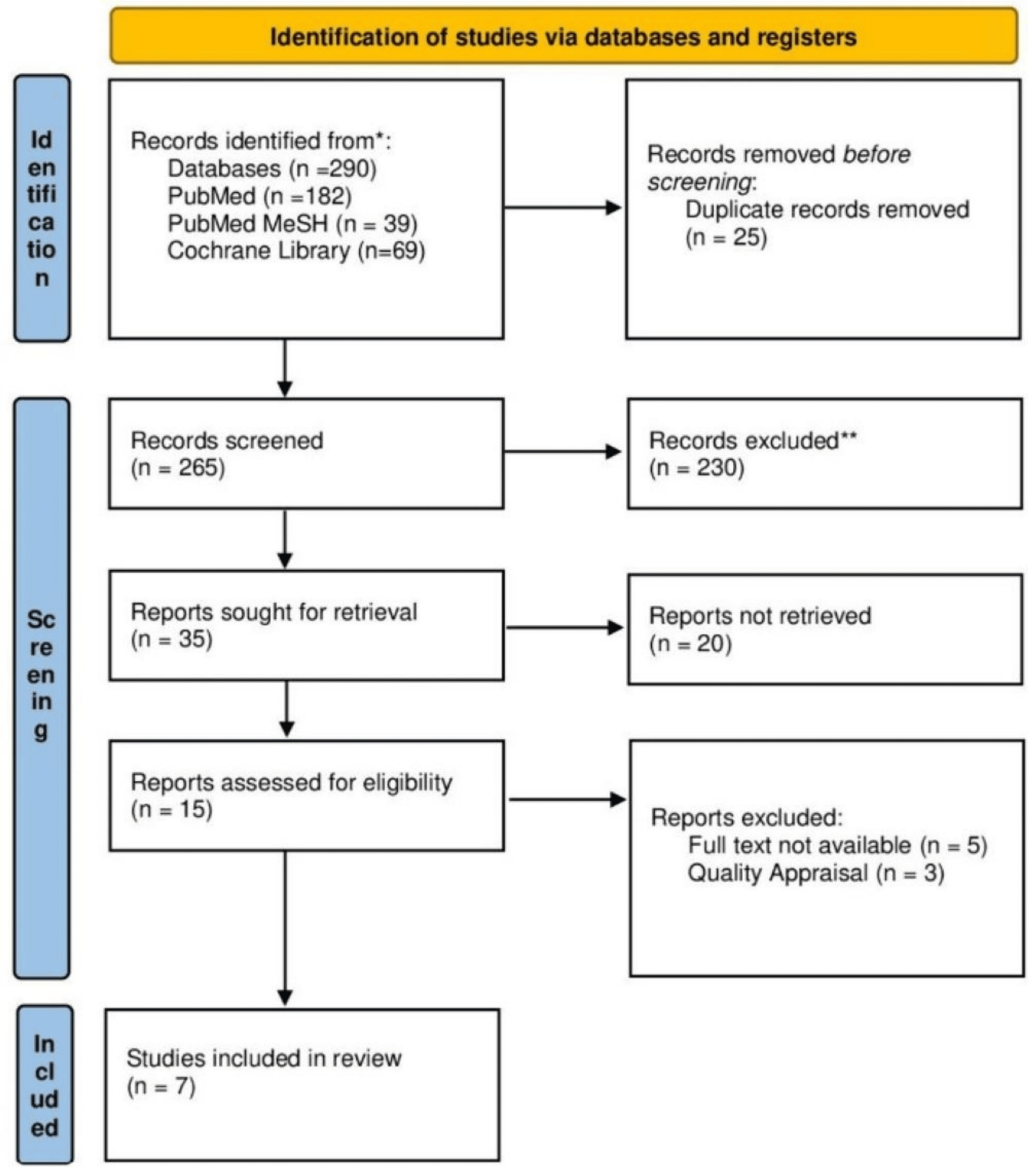
PRISMA flowchart showing the process of article selection. PRISMA: Preferred Reporting Items for Systemic Review and Meta-Analysis.

We were able to find a total of 290 suitable articles across the various databases, of which 25 duplicates were initially eliminated. After thoroughly screening the articles by reviewing their titles and abstracts and retrieving the full texts, 15 articles were shortlisted. These full-text articles were assessed for eligibility and quality, leading to the final selection of seven articles for review. The study selection process is illustrated in Figure 1 on the PRISMA flowchart.

The articles were assessed for eligibility using the relevant quality appraisal tools. Table 2 shows the results of the quality appraisal.

Study Characteristics

Of the seven studies included in this review, six were observational studies, and one was a randomized control trial. The selected studies have been summarized in Table 2, including the type of study it is and important points to note. The table provides the author's name, study design, and salient points of the references cited in the review [17]. 

**Table 2:** List of references utilized in the review. The table provides the author's name, study design, and salient points of the references cited in the review. ERAS: Enhanced Recovery After Surgery, LOS: length of stay, A&F: audit and feedback.

Authors and year of publication	Type of study	Salient points
Olson et al. 2021 [7]	Retrospective cohort design	An identified subset of ERAS elements most influential on LOS and readmission following colorectal surgery.
Choi et al. 2022 [13]	Retrospective cohort design	Describes the execution and improvement of ERAS protocols through a multidisciplinary team approach.
Pagano et al. 2021 [18]	Cluster randomized trial	Audit and feedback approach (A&F) was adopted alongside a cluster randomized trial to estimate the true impact of the ERAS protocol on a large population
Yu et al. 2022 [19]	Retrospective cohort design	Group practice based on a modified ERAS protocol shortens postoperative hospital stays with fewer morbidities as compared to solo practices.
Zorrilla-Vaca et al. 2021 [20]	Prospective cohort design	Highlights the importance of strong leadership, experience and setting up a multidisciplinary team when developing an ERAS protocol for colorectal surgery.
Milone et al. 2022 [21]	Cohort, prospective	The primary goal was to associate the percentage of ERAS adherence to functional recovery after minimally invasive colorectal cancer surgery.
Norman et al. 2020 [22]	Interrupted time series	Evaluated the sustainability of the ERAS program after supervision of guideline compliance was eliminated.

Discussion

In 2008, a protocol similar to the ERAS was developed called the FAST TRACK strategy, which included early feeding, ambulation, and gastrointestinal (GI) motility drugs. However, the program was discontinued due to the absence of essential personnel, such as coordinators needed to establish ERAS and a lack of academic interest in studying the effects of ERAS implementation. When the ERAS protocol was to be adopted by this program, there was a turnaround with the implementation of a performance improvement team, which consisted of various professionals, including surgeons, anesthesiologists, project coordinators, ward nurses, nutritionists, sports therapists, administrative staff in insurance, and engineers. A series of meetings were held to apprise the team members of the latest knowledge, protocols, and education tools to ensure compliance measurement completion. After applying the ERAS protocol, continuous upgrades were made based on feedback and monitoring. Audit meetings were carried out for four to six months. It was observed that the ERAS protocol reduced inflammation rates and reduced hospital stays [13].

According to the study performed by Choi et al. [13], a multidisciplinary committee that includes a performance improvement team is essential for a successful implementation of ERAS, with a goal and purpose clearly stated and each member's role identified. Various patient educational materials were developed, including the standard prescription and various compliance measurement tools, favoring better compliance. A team approach was adopted too, as it was acknowledged that the difficulty reaching an agreement failed the previous 'fast track’. For continual implementation, one of the most important steps is checking the compliance of each protocol item. Compliance audits were conducted regularly to evaluate the compliance of each component. Some areas of improvement were also identified. As real-time compliance checks were not feasible, the data analysis was conducted retrospectively; hence, its findings cannot be applied immediately to patient care. A patient-centered approach was adopted leveraging a wearable device that recorded the patient's steps, exercise distance, and calorie intake in real time to check for items with poor compliance. Based on the measurements, adjustment was made accordingly to improve adherence significantly [13].

A study performed by Pagano et al. [18] in Italy found that for successful implementation of the ERAS protocol, there was a need for three key elements. (1) An up-to-date and shared ERAS operating protocol. (2) Establishing a specialized team responsible for training personnel and ensuring adherence to the protocol. (3) An audit and feedback (A&F) system was created to ensure compliance with the protocol and track clinical outcomes. A newsletter was also sent out bi-monthly to all the ERAS teams to sustain involvement and enthusiasm and share information on the study progress and relevant news. The A&F system was accessible to the professionals involved, and it displayed various graphs representing the adherence rate to all the ERAS items to identify critical issues and promptly address them with corrective actions [18].

In a study performed by Yu et al. [19] in China comparing ERAS compliance in a solo practice vs. group practice, it was found that the compliance rate was higher in group practice than in solo practice. The adherence rate of implementers has been pointed out as a key success factor in the implementation of ERAS. It was found that some ERAS elements were difficult to adhere to by traditionally trained surgeons as they might give medical orders during ward rounds based on experience and habits, which may account for the lower recovery rate of patients in solo group practice. The attending surgeons commonly work with patients operated on by other group practice members, during which they give medical orders based on the ERAS checklist, further increasing compliance [19]. 

In a study performed by Norman et al. [22], there was a structured implementation pathway that resulted in an adherence rate of >80% that was developed and shared with all participating healthcare professionals through various field training and internal training courses crucial to uphold the clinical basis of the program and to adapt this evidence contained in the ERAS guidelines to the local clinical reality. As problems were identified, they were addressed as time elapsed between one training course and the other, strengthening the adherence rate. It has been shown that the post-operative component of ERAS is the least adhered to even after structured implementation. The failure to adhere to the intraoperative goal-directed fluid management aspect of ERAS has been associated with tissue edema and other inflammation, which increases the risk of anastomotic leakage. The increase in the adherence rate in this program resulted in a significant reduction in the overall length of stay compared to the national average, with a net difference of three days and a reduced readmission rate of 5.1% from the average reported rate of 7% [22].

Olson et al. [7] also conducted a study to assess the element of ERAS associated with decreased LOS and readmission rates. This study was carried out to address the difficulty in adhering to every component of ERAS. A positive correlation was found between readmission and the average morphine milligram equivalent (MME) per day, with an OR of 1.06 per unit increase in MME. Patients with minimally invasive procedures placed on multimodal pain regimes and <16 MME per day had lesser LOS. Using various machine learning tools to analyze the result of the multiple elements of ERAS, it was found that complete preop bowel prep (OR=0.115, p=0.0027), multimodal pain regimen (OR=0.14, p<0.0001), and mobilization within 24 hours of the procedure (OR=14.8, p<0.0001) were protective. For readmission risk, <60 MME/day, with over 15 elements of ERAS implemented and the use of postop oral fluid intake at four hours, were associated with the lowest risk of readmission. Also, early mobilization 24 h after surgery and strict glucose control, glucose levels <180 mg/dl were associated with low readmission rates. This supports the minimization of MMEs and the adoption of multimodal pain management, encouraging reduced narcotics usage [7].

Zorrilla-Vaca et al. [20] studied various institutional factors associated with adherence to ERAS protocols. It was found that scheduled multidisciplinary meetings and program duration were facilitators of adherence to ERAS guidelines. In contrast, large case volumes and numerous anesthesiologists can hinder adherence to ERAS guidelines. Achieving standardized healthcare delivery can be challenging in high institutions due to difficulties in ensuring a consensus among the employees. The size of the hospital could also translate to excessive workload, which is a crucial determinant of a lack of adherence to clinical guidelines. Also, larger staff sizes increase the variability of individual perspectives and experiences. Adherence can be promoted using either interactive, e.g., visual feedback tool, which displays a color-coded score of compliance, or purely academic tools through continuing medical education such as a simulation-based care pathways training curriculum. Another potential method includes patient engagement using mobile device applications, which has been shown to reduce the workload for the medical team. Various studies have addressed the importance of teamwork with regular multidisciplinary meetings, which will improve communication across multiple disciplines alongside program duration, which is a surrogate for clinical experience [20].

Milone et al. [21] attempted to address the ways to simplify the ERAS component to facilitate its penetrance into clinical practice since full adherence (100%) is not possible. In this study, it was noted that there was no clinical difference in patient outcome between a 100% and >75% adherence rate, for which analysis of the different items of ERAS component was performed to reveal a clear association on which item in the protocol has an impact on patient's recovery. With difficulty in full adherence to all aspects of ERAS, some authors have developed a simplified ERAS protocol known as remove, ambulate, post-operative analgesia, introducing diet (RAPID), which suggests the removal of nasogastric tubes at the end of the intervention, early mobilization, introducing oral fluids and diet early, reduction of pain management with opioids, removal of a urinary catheter, and discontinuation of intravenous fluids at the third postoperative day [21].

In a study by Norman et al. [22], the absence of supervision decreased compliance with the ERAS protocol, particularly the post-operative component. Intravenous fluid discontinuation on postoperative day 1 (POD1), administration of clear fluids on postoperative day 0 (POD0), mobilization on POD1, and post-operative gum chewing. It was found that the shifting culture in health units presented significant barriers to behavioral change. Interviews and focus groups were used to assess the status quo and develop solutions to overcome barriers in collaboration with the front-line staff. In addition, the development of an electronic visual management system was also envisaged to help promote adherence [22].

In a study performed by Catarci et al. [23], there was a structured implementation pathway that resulted in an adherence rate of >80% that was developed and shared with all participating healthcare professionals through various field training and internal training courses crucial to uphold the clinical basis of the program and to adapt this evidence contained in the ERAS guidelines to the local clinical reality. As problems were identified, they were addressed as time elapsed between one training course and the other, strengthening the adherence rate. It has been shown that the post-operative component of ERAS is the least adhered to even after structured implementation. The failure to adhere to the intraoperative goal-directed fluid management aspect of ERAS has been associated with tissue edema and other inflammation, which increases the risk of anastomotic leakage. The increase in the adherence rate in this program resulted in a significant reduction in the overall length of stay compared to the national average, with a net difference of three days and a reduced readmission rate of 5.1% from the average reported rate of 7% [23].

After rigorous screening, this is the first systematic review of various barriers to implementing the ERAS protocol. There was a certain limitation to the paper, such as the paucity of information about the implementation process and possible core components of ERAS, which have been associated with better outcomes. This research also speaks to possible methods to ensure adherence to various protocols arising in the field of medicine.

## Conclusions

The ERAS protocol has been shown to allow for quick post-operative recovery. However, successful implementation requires setting goals based on established criteria and modifying them to suit the peculiarities of each hospital. This necessitates a well-informed team with proficient and updated knowledge of the ERAS protocol, with a clear delineation of roles, collaboration, and education that should be implemented for each job within the team. In addition, continuous modification and post-implementation research is necessary for refining and expanding the protocol. Group practice can also foster adherence to the ERAS protocol and countering old habits and tendencies of the traditional solo surgical approach. 

Identifying various aspects of ERAS associated with better recovery is particularly important in small community programs where implementation barriers are most likely to exist. It may enable surgeons lacking well-organized support systems to focus their efforts on implementing the various subsets of ERAS protocol that have been shown to have the greatest impact on patient outcomes.
